# Effectiveness of a behavioural intervention delivered by text messages (safetxt) on sexually transmitted reinfections in people aged 16-24 years: randomised controlled trial

**DOI:** 10.1136/bmj-2022-070351

**Published:** 2022-09-28

**Authors:** Caroline Free, Melissa J Palmer, Ona L McCarthy, Lauren Jerome, Sima Berendes, Megan Knight, James R Carpenter, Tim P Morris, Zahra Jamal, Farandeep Dhaliwal, Rebecca S French, Ford Colin Ian Hickson, Anasztazia Gubijev, Kaye Wellings, Paula Baraitser, Ian Roberts, Julia V Bailey, Tim Clayton, Karen Devries, Phil Edwards, Graham Hart, Susan Michie, Louis Macgregor, Katy M E Turner, Kimberley Potter

**Affiliations:** 1 Clinical Trials Unit, Department of Population Health, London School of Hygiene and Tropical Medicine, London, UK; 2 Clinical Trials Unit, Department of Medical Statistics, London School of Hygiene and Tropical Medicine, London, UK,; 3 MRC Clinical Trials Unit, London, UK; 4 Department of Public Health, Environments and Society, London School of Hygiene and Tropical Medicine, London, UK; 5 Bristol Veterinary School, University of Bristol, Bristol, UK; 6 Centre for Global Health, King’s College London, London, UK; 7 Sigma Research, London School of Hygiene and Tropical Medicine, London, UK; 8 eHealth Unit, Research Department of Primary care and Population Health, University College London, London, UK; 9 Department of Infection and Population Health, University College London, London, UK; 10 Centre for Outcomes Research and Effectiveness, University College London, London, UK; 11 Department of Global Health and Development, London School of Hygiene and Tropical Medicine, London, UK; 12 Department of Social Science, University College London Institute of Education, London, UK

## Abstract

**Objective:**

To quantify the effects of a series of text messages (safetxt) delivered in the community on incidence of chlamydia and gonorrhoea reinfection at one year in people aged 16-24 years.

**Design:**

Parallel group randomised controlled trial.

**Setting:**

92 sexual health clinics in the United Kingdom.

**Participants:**

People aged 16-24 years with a diagnosis of, or treatment for, chlamydia, gonorrhoea, or non-specific urethritis in the past two weeks who owned a mobile phone.

**Interventions:**

3123 participants assigned to the safetxt intervention received a series of text messages to improve sex behaviours: four texts daily for days 1-3, one or two daily for days 4-28, two or three weekly for month 2, and 2-5 monthly for months 3-12. 3125 control participants received a monthly text message for one year asking for any change to postal or email address. It was hypothesised that safetxt would reduce the risk of chlamydia and gonorrhoea reinfection at one year by improving three key safer sex behaviours: partner notification at one month, condom use, and sexually transmitted infection testing before unprotected sex with a new partner. Care providers and outcome assessors were blind to allocation.

**Main outcome measures:**

The primary outcome was the cumulative incidence of chlamydia or gonorrhoea reinfection at one year, assessed by nucleic acid amplification tests. Safety outcomes were self-reported road traffic incidents and partner violence. All analyses were by intention to treat.

**Results:**

6248 of 20 476 people assessed for eligibility between 1 April 2016 and 23 November 2018 were randomised. Primary outcome data were available for 4675/6248 (74.8%). At one year, the cumulative incidence of chlamydia or gonorrhoea reinfection was 22.2% (693/3123) in the safetxt arm versus 20.3% (633/3125) in the control arm (odds ratio 1.13, 95% confidence interval 0.98 to 1.31). The number needed to harm was 64 (95% confidence interval number needed to benefit 334 to ∞ to number needed to harm 24) The risk of road traffic incidents and partner violence was similar between the groups.

**Conclusions:**

The safetxt intervention did not reduce chlamydia and gonorrhoea reinfections at one year in people aged 16-24 years. More reinfections occurred in the safetxt group. The results highlight the need for rigorous evaluation of health communication interventions.

**Trial registration:**

ISRCTN registry ISRCTN64390461.

## Introduction

The burden of sexually transmitted infections (STIs) such as chlamydia and gonorrhoea is highest in people aged 16-24 years.^1^ ^2^ Limited knowledge of risk reduction strategies and poor sexual communication skills might contribute to this increased risk.^3^ ^4^

Health communications delivered by text message are effective, cheap, and highly cost effective for some behaviours, such as smoking cessation.^5^ ^6^ The World Health Organization currently recommends the use of digital health communication for strengthening health systems, including for sexual and reproductive health, provided that privacy and sensitivity concerns can be taken into consideration.^7^ The covid-19 pandemic has led to an expansion in the use of digital technologies, including text messages supporting healthcare systems. Current guidance and practice reflect an assumption that provided privacy and sensitivity concerns are considered, digital health communication using text messages poses no risk of harm.^7^

We developed an intervention, safetxt, delivered by text messages to improve safer sex behaviours in people aged 16-24 with chlamydia or gonorrhoea. In qualitative interviews and a pilot trial with 200 participants, we found that our interactive support via text message was acceptable, and reportedly altered the behaviours targeted. The trial methods were feasible. A main trial to establish the effect on STIs was warranted.^8^ ^9^

## Methods

We quantified the effects of the safetxt intervention on chlamydia and gonorrhoea reinfection at one year and hypothesised that safetxt would reduce the risk of both at one year and improve three key safer sex behaviours: partner notification at one month, condom use, and STI testing before unprotected sex with a new partner.

In this parallel group individual level randomised superiority trial, care providers and outcome assessors were blind to allocation. Participants were recruited from 92 sexual health clinics in the United Kingdom, and the intervention was delivered in the community by mobile phone. Our methods were prespecified and are published in the trial protocol.^10^

Eligible participants were aged 16-24 years, owned a mobile phone, were able to provide informed consent, and either had a diagnosis of or had started treatment for chlamydia or gonorrhoea or non-specific urethritis in the past two weeks. We excluded those known to be a sexual partner of someone already recruited to the trial. Participants provided written informed consent in person or via the trial enrolment website.

### Randomisation and masking

An automated, independent, computer based system remote from the recruiting sites generated the randomisation sequence. Automated links between the web based enrolment randomisation system and system sending intervention and control group messages ensured allocation concealment. An information technologist with no role in research aspects of the trial monitored all systems. Laboratory staff were masked to treatment. The statisticians were masked to treatment allocation until the code was broken after the main analysis. Owing to the nature of the intervention, participants could surmise their allocated treatment.

### Procedures

Safetxt aimed to reduce chlamydia and gonorrhoea reinfection by encouraging participants to correctly follow instructions for STI treatment, including informing partners about their own infection, promoting condom use, and encouraging participants to seek STI testing before unprotected sex with a new partner.^8^ Safetxt was developed based on the COM-B (capability, opportunity, motivation, and behaviour) model and evidence on factors that influence behaviours. To ensure that the intervention was acceptable and accessible, we shaped the content based on the views of 64 users who varied by gender, sexual orientation, sociodemographic background, ethnicity, and area of residence. The content that promoted condom use and STI testing was informed by existing face-to-face interventions shown to increase condom use and reduce STIs. Safetxt uses a novel approach to support partner notification by providing non-blaming and non-stigmatising information and examples of how others, in a range of relationships, told their partners about an infection. Safetxt comprises educational, enabling, and incentivising behaviour change strategies and 12 evidence based behaviour change techniques: information about the health consequences of behaviour, instruction on how to carry out the behaviour, demonstrations of risk reduction behaviour, social support, emotional support, social rewards, non-specific incentives, encouragement to add objects to the environment to trigger behaviours, anticipated regret, problem solving, action planning techniques, and reframing.^11^ The information on safer sexual practices was in accordance with existing guidelines.^12^ The messages were tailored according to sex or gender and sexual orientation. All participants who have sex with men received messages about how others had negotiated condom use. Women and those who have sex with women were sent messages about emergency contraception. Men who have sex with men were sent messages about HIV post-exposure prophylaxis. Women who only have sex with women were not sent messages about condom use. The information provided was specific to the STI diagnosed. This tailoring resulted in different numbers of messages being sent to those of different sex or gender and sexual orientation.

The core message sets included 42 messages for women who have sex with women, 74 for women who have sex with men or with men and women, 69 for men who have sex with women, 76 for men who have sex with men, and 79 for men who have sex with men and women. Recipients could request additional messages on specific topics. Participants were sent text messages starting on the day of randomisation: four texts daily for days 1-3, one or two daily for days 4-28, two or three weekly for month 2, and 2-5 monthly for months 3-12 (see examples of messages in appendix 1).

Participants in the control group received a monthly text message asking for any changes to their postal or email address. All participants received usual care and were free to seek any other existing services or support.

The text messages were sent automatically. Participants were able to stop the messages or set times when they did not want to receive them. Self-reported outcomes were assessed at one and 12 months by postal paper based questionnaire or the trial web based data entry form. At 12 months, chlamydia and gonorrhoea infections were assessed by nucleic acid amplification test using self-sampling postal kits. One accredited laboratory assessed the postal tests at 12 months. Chlamydia and gonorrhoea reinfections during 12 months’ follow-up were assessed by checking the records of clinics where participants reported they had completed tests.

### Outcomes

The primary outcome was the incidence of chlamydia or gonorrhoea reinfection at one year. Secondary outcomes at four weeks were informing the last sexual partner before the test to seek treatment, clinic attendance by partner for treatment, taking prescribed antibiotics and avoiding sex for seven days after treatment, and condom use at last sexual encounter. Intermediate outcomes at four weeks were knowledge related to STIs (the consequences of behaviour and how to avoid infection), attitude towards notification of partners, and self-efficacy about correct condom use, negotiating condom use, and telling a partner about an infection. Secondary outcomes at one year were STI diagnosis after joining the trial (self-report confirmed by postal test results and clinic records), condom use at last sexual encounter, STI self-testing before sex with most recent new partner (self-reported and confirmed by clinic record of a test), sex with someone new since joining the trial, condom use at first sexual encounter with someone new, participants’ report that the last new partner was tested for STI before having sex with them, and number of sexual partners since joining the trial. Process outcomes at one year were reading and sharing of intervention content, number of text messages read, whether anyone else read the messages, and, if yes, how the participant felt about the messages being read, and reading someone else’s messages in the trial (control group) and someone else in the trial reading the participant’s messages (intervention group). Data on adverse events were collected on experience of partner violence and road traffic incidents when the participant was the driver in the past year (as road traffic incidents are a known harm of mobile phone use if used whilst driving).

### Statistical analyses

Assuming an event rate for the cumulative incidence of chlamydia or gonorrhoea of 20%,^8^ ^13^ the trial was designed to detect a reduction in chlamydia or gonorrhoea reinfection from 20% to 16% (relative risk 0.8). To detect this difference a trial with 5000 participants would have 90% power using an α level of 0.05. The sample size calculation allows for 2% of participants in the control arm viewing the intervention messages (as seen in the pilot study) and up to 20% losses to follow-up. The trial steering committee reviewed the (masked) event rate after 546 patients had completed 12 months’ follow-up, and it recommended an increase in the sample size to 6250 because of a lower than expected event rate of 15.6%.

The primary analysis was by intention to treat. A detailed statistical analysis plan was published on the trial website before analysis and unblinding.^14^ For the primary outcome we compared the cumulative incidence of chlamydia or gonorrhoea reinfection at one year in each group using logistic regression. We used multiple imputation by chained equations (MICE) using the predictors of the outcome identified in the baseline data and in four week data to impute one year outcome data.^15^ We adjusted the primary analysis regression for the prespecified baseline covariates (age, type of STI at baseline, sex or gender, sexual orientation, and ethnicity).^16^ We report the adjusted odds ratios with 95% confidence intervals. The analysis of the secondary outcomes was similar to the analysis of the primary outcome. A complete case analysis was conducted as a supplementary analysis. We conducted prespecified subgroup analysis on the imputed dataset for age (16-19 years; 20-24 years), sex or gender (female-woman; male-man), sex or gender sexual orientation (men who have sex with men, and men who have sex with men and women; men who have sex with women; women who have sex with men, and women who have sex with men and women), ethnic group (white British/other white ethnicity; black/black British; all other groups), and adjusted indices of multiple deprivation^17^ (first and second fifths (least deprived), third fifth, and fourth and fifth fifths (most deprived)). Across the subgroups, we assessed heterogeneity of treatment effect and estimated odds ratios with 99% confidence intervals.^14^ ^18^ For the intermediate outcomes, we carried out a complete case analysis and compared the summed scores using a linear regression. We conducted structural equation modelling using the latent variable intermediate outcomes derived from confirmatory factor analysis for the intermediate outcomes (see appendix 2 for full details). All analyses were done using Stata v 15.1. This study had no data monitoring committee.

### Patient and public involvement

Patients and members of the public were involved in all phases of this study. Before developing the intervention, we discussed possible safer sex interventions in five discussion groups with people aged 16-24 years based in Southwark Further Education College (total 25 participants). The students were enthusiastic about receiving information and support via mobile phone. We worked with patients, who were recruited as research participants in focus groups, to design the content of the intervention.^8^ We met with 14 patient representatives from King’s College Hospital Sexual and Reproductive Health user group who helped design the patient information, questionnaires, and consent and follow-up procedures. A patient representative was included in the trial steering committee. A group of patient representatives are actively involved in disseminating the trial results.

## Results

**Fig 1:**
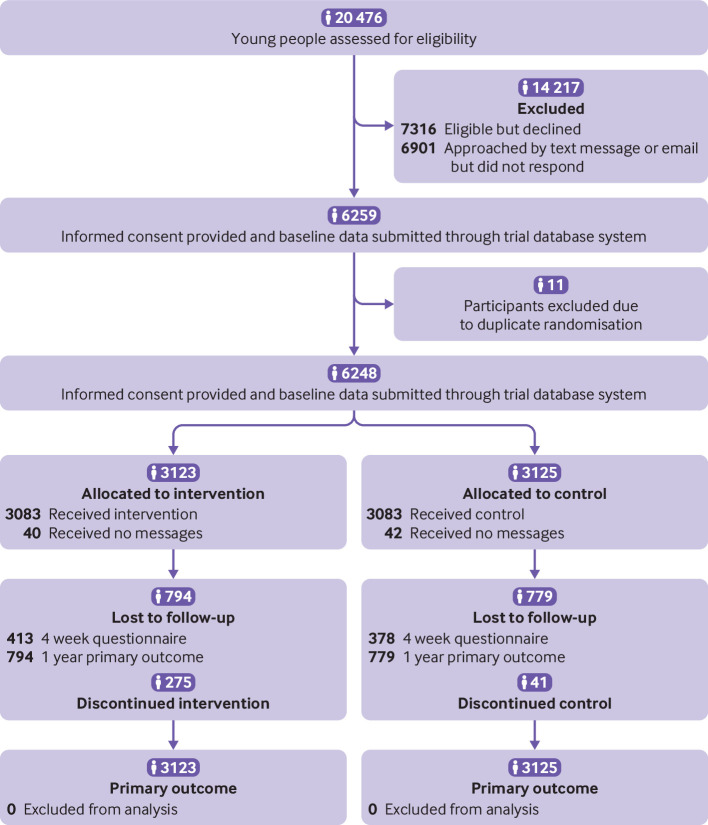
Consolidated standards of reporting trials (CONSORT) diagram

Between 1 April 2016 and 23 November 2018, we assessed 20 476 young people for eligibility. Of these, we excluded 14 217 before randomisation (7316 were not eligible and 6901 were eligible and approached by text message or email but did not respond) (fig 1). Informed consent was provided, and baseline data for 6259 participants was submitted through the trial database system. Eleven participants were excluded owing to duplicate randomisations. Of the remaining 6248 participants, 3123 were randomised to the intervention arm and 3125 to the control arm. Overall, 281/6248 (4.5%) participants withdrew from the trial before follow-up: 134/3123 (4.3%) in the intervention group and 147/3125 (4.7%) in the control group. A total of 4675 (74.8%) participants provided data for the primary outcome (safetxt: 2329/3123 (74.6%); control: 2346/3125 (75.0%)). All participants were included in the intention-to-treat analysis, with missing data imputed using MICE. A total of 2167/2412 (89.8% of the intervention group) respondents read all or most of the messages (2167/3125 (69.3%) of the intervention group).

**Fig 2:**
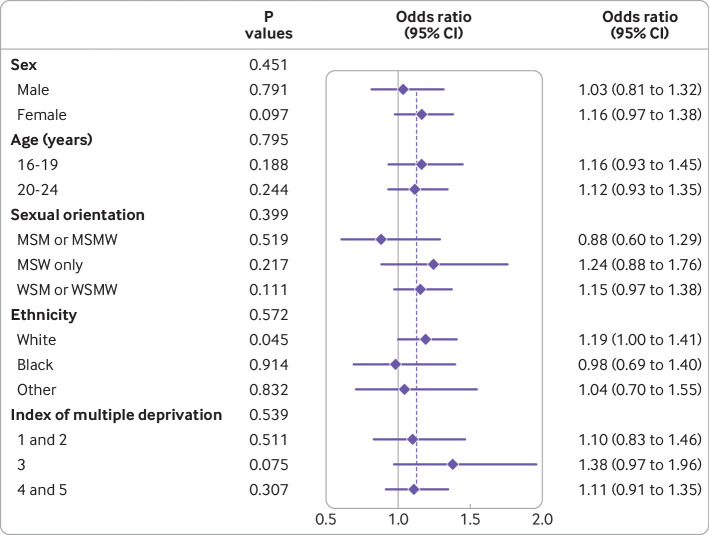
Primary outcome by prespecified subgroups. MSM=men who have sex with men; MSMW=men who have sex with men and women; MSW=men who have sex with women; WSM=women who have sex with men; WSMW=women who have sex with men and women

Baseline sociodemographic characteristics were similar between the groups (table 1). Table 2 shows the results for the primary and secondary outcomes. The cumulative incidence of chlamydia or gonorrhoea reinfection was 22.2% (693/3123) in the intervention arm versus 20.3% (633/3125) in the control arm (odds ratio 1.13, 95% confidence interval 0.98 to1.31, P=0.08). When only those participants with complete primary outcome data (4675/6248) were included in the primary analysis model, the odds ratio was 1.14 (0.98 to 1.31, P=0.08). In a per protocol analysis that was not prespecified, the corresponding intervention effect was 1.17 (0.99 to 1.38, P=0.06, see appendix 2, table S3). We found no evidence to suggest that the effect of the intervention was different among participants in any of the prespecified subgroups (fig 2).

**Table 1:** Baseline characteristics of participants with a history of chlamydia, gonorrhoea, or non-specific urethritis assigned to a series of text messages to improve sexual health (safetxt intervention) or to text messages querying change of address (control group). Values are numbers (percentages) unless stated otherwise

Characteristics	Safetxt group (n=3123)	Control group (n=3125)
**Age group (years)**		
16-19	1189 (38.1)	1117 (35.7)
20-24	1934 (61.9)	2008 (64.3)
Mean (SD) age (years) (based on integer)	20.3 (2.1)	20.4 (2.1)
**Gender**		
Female	2047 (65.5)	2020 (64.6)
Male	1065 (34.1)	1097 (35.1)
Non-binary	11 (0.4)	8 (0.3)
**Ethnicity**		
White British/other white	2428 (77.7)	2436 (78.0)
Black/black British-Caribbean, African, other	380 (12.2)	347 (11.1)
Asian/Asian British-Bangladeshi, Chinese, Indian, Pakistani, other	89 (2.8)	91 (2.9)
Mixed	174 (5.6)	205 (6.6)
Other	52 (1.7)	46 (1.5)
Index of multiple deprivation fifth*	n=3099	n=3096
1st (least deprived)	439 (14.2)	424 (13.7)
2nd	516 (16.7)	527 (17.0)
3rd	608 (19.6)	590 (19.1)
4th	768 (24.8)	761 (24.6)
5th (most deprived)	768 (24.8)	794 (25.6)
**Educational level†**	n=2996	n=2990
Primary and secondary (age ≤16 years)	436 (14.6)	450 (15.1)
Secondary onwards (age ≥17 years)	1352 (45.1)	1348 (45.1)
Still in full time education	1208 (40.3)	1192 (39.9)
**Gender and sexual orientation**		
Women who have sex with men only	1901 (60.9)	1855 (59.4)
Men who have sex with women only	790 (25.3)	778 (24.9)
Women who have sex with women only	20 (0.6)	17 (0.5)
Men who have sex with men only	226 (7.2)	258 (8.3)
Women who have sex with women and men	125 (4.0)	147 (4.7)
Men who have sex with women and men	49 (1.6)	60 (1.9)
Those with non-binary gender who have sex with men	7 (0.2)	3 (0.1)
Those with non-binary gender who have sex with women	1 (0)	2 (0.1)
Those with non-binary gender who have sex with women and men	3 (0.1)	3 (0.1)
Not stated	1 (0)	2 (0.1)
**Baseline diagnosis**		
Chlamydia	2449 (78.4)	2433 (77.9)
Gonorrhoea	283 (9.1)	303 (9.7)
Gonorrhoea and chlamydia	159 (5.1)	155 (5.0)
Gonorrhoea or non-specific urethritis	27 (0.9)	32 (1.0)
Non-specific urethritis	125 (4.0)	123 (3.9)
Unknown	80 (2.6)	79 (2.5)
**Condom used during last sexual encounter**		
Yes	747 (23.9)	806 (25.8)
No	2314 (74.1)	2273 (72.7)
Unsure	62 (2.0)	46 (1.5)
**Condom used during first sexual encounter with last new partner**		
Yes	981 (31.4)	1035 (33.1)
No	2065 (66.1)	2010 (64.3)
Unsure	77 (2.5)	80 (2.6)
**Tested before sex with last new partner**		
Yes	1242 (39.8)	1243 (39.8)
No	1798 (57.6)	1787 (57.2)
Unsure	83 (2.7)	95 (3)
**Partner tested before sex with last new partner**	n=3120	n=3125
Yes	437 (14)	457 (14.6)
No	1189 (38.1)	1181 (37.8)
Unsure	1494 (47.9)	1487 (47.6)
**No of partners in past 12 months**	n=3120	n=3122
0	5 (0.2)	2 (0.1)
1	496 (15.9)	538 (17.2)
≥2	2619 (83.9)	2582 (82.7)

* Reduced denominator—index of multiple deprivation fifth was missing for some participants who provided an invalid postcode.

† Reduced denominator—education information was missing for some participants due to non-response.

**Table 2:** Primary and secondary outcomes in participants with a history of chlamydia, gonorrhoea, or non-specific urethritis assigned to a series of text messages to improve sexual health (safetxt intervention) or to text messages querying change of address (control group). Values are numbers (percentages) estimated from imputed data

Outcomes	Safetxt group (n=3123)	Control group (n=3125)	Odds ratio (95% CI)	P value
**Primary outcome (1 year)**				
Cumulative incidence of chlamydia or gonorrhoea reinfection	693 (22.2)	633 (20.3)	1.13 (0.98 to 1.31)	0.09
**Secondary outcomes (4 weeks)**				
Correctly treated for STI (took prescribed antibiotics and avoided sex for 7 days after treatment)	2798 (89.6)	2769 (88.6)	1.11 (0.94 to 1.32)	0.22
Participant told last partner they had sex with before testing positive to get treatment	2673 (85.6)	2625 (84.0)	1.14 (0.99 to 1.33)	0.08
Partner attended clinic for treatment (identified from clinic records)	365 (11.7)	406 (13.0)	0.88 (0.75 to 1.02)	0.10
Condom use at last sexual encounter	1312 (42.0)	1238 (39.6)	1.12 (1.00 to 1.25)	0.05
**Secondary outcomes (1 year)**				
Condom use at last sexual encounter	1056 (33.8)	975 (31.2)	1.14 (1.01 to 1.28)	0.04
≥2 sexual partners since joining the trial	1777 (56.9)	1713 (54.8)	1.11 (1.00 to 1.24)	0.06
Sex with someone new since joining the trial	2177 (69.7)	2106 (67.4)	1.13 (1.00 to 1.28)	0.06
Condom use at first sex with most recent new partner	1699 (54.4)	1522 (48.7)	1.27 (1.11 to 1.45)	0.001
STI testing for self, before first sexual encounter with most recent new partner (self-reported)	2067 (66.2)	2128 (68.1)	0.92 (0.79 to 1.06)	0.24
STI testing for self, before first sexual encounter with most recent new partner (testing confirmed by clinic record)	1234 (39.5)	1278 (40.9)	0.95 (0.82 to 1.10)	0.48
Most recent new partner was tested for STI before sex with participant	977 (31.3)	881 (28.2)	1.15 (0.88 to 1.51)	0.28
Road traffic incident in past year when participant was driver	106 (3.4)	100 (3.2)	1.05 (0.76 to 1.47)	
Experience of partner violence in past year	103 (3.3)	103 (3.3)	1.01 (0.75 to 1.38)	
Diagnosis of “any” STI after joining trial according to postal test results and clinic records	693 (22.2)	647 (20.7)	1.10 (0.95 to 1.29)	0.21

STI=sexually transmitted infection.

Analyses based on intention-to-treat principle; logistic regression analysis adjusted for prespecified baseline covariates (age, type of STI at baseline, sexual orientation, and ethnicity).

At four weeks, 85.6% (2673/3123) of participants in the intervention arm versus 84.0% (2625/3125) in the control arm had notified the last partner they had sex with before testing positive to get treatment (odds ratio 1.14, 95% confidence interval 0.99 to 1.33, P=0.08), 89.6% (2798/3123) in the intervention arm versus 88.6% (2769/3125) in the control arm followed the correct treatment for STIs (1.11, 0.94 to 1.32, P=0.22), and, according to data from clinics, the partners of 11.7% (365/3123) of participants in the intervention arm versus 13.0% (406/3125) in the control arm attended for treatment (0.88, 0.75 to 1.02, P=0.10) (table 2). At four weeks, 42.0% (1312/3123) of participants in the intervention arm versus 39.6% (1238/3125) in the control arm (1.12, 1.00 to 1.25, P=0.05) reported using a condom at last sexual encounter (table 2). This difference was sustained at 12 months (33.8% (1056/3123) intervention *v* 31.2% (975/3125) control, 1.14, 1.01 to 1.28, P=0.04). At one year, 54.4% (1699/3123) of participants in the intervention arm versus 48.7% (1522/3125) in the control arm reported using a condom at first sexual encounter with their most recent new partner (1.27, 1.11 to 1.45, P=0.001) (table 2). No difference was found in participants testing before sex with a new partner according to self-report (66.2% (2067/3123) intervention *v* 68.1% (2128/3125) control, 0.92, 0.79 to 1.06, P=0.24) or self-report confirmed by clinic test (39.5% (1234/3123) intervention *v* 40.9% (1278/3125) control, 0.95, 0.82 to 1.10, P=0.48). The most recent new partner of 31.3% (977/3123) of participants in the intervention arm versus 28.2% (881/3125) in the control arm was tested for an STI before sex with the participant (1.15, 0.88 to 1.51, P=0.28) (table 2). Since joining the trial, 56.9% (1777/3123) of participants in the intervention arm versus 54.8% (1713/3125) in the control arm reported having two or more partners (1.11, 1.00 to 1.24, P=0.06), and 69.7% (2177/3123) in the intervention arm versus 67.4% (2106/3125) in the control arm reported having sex with someone new (1.13, 1.00 to 1.28, P=0.06) (table 2). The diagnosis of any STI was reported in 22.2% (693/3123) of participants the intervention arm versus 20.7% (647/3125) in the control arm (1.10, 0.95 to 1.29, P=0.21) (table 2). Self-reported partner violence or road traffic incidents were similar between the groups. Table 3 shows intermediate and process outcomes (also see tables S1 and S2 in appendix 2). The intervention was associated with small increases in the intermediate outcomes: knowledge related to STIs (coefficient 0.10, P=0.04) and correct condom use self-efficacy (0.32, P<0.001).

**Table 3:** Intermediate and process outcomes in participants with a history of chlamydia, gonorrhoea, or non-specific urethritis assigned to a series of text messages to improve sexual health (safetxt intervention) or to text messages querying change of address (control group). Values are numbers (percentages) unless stated otherwise

Outcomes	Safetxt group	Control group	Coefficient* (95% CI)	P value
**Mean (SD) intermediate outcomes (summed items)**	n=2656	n=2705		
Knowledge related to STIs	12.38 (1.84)	12.29 (1.84)	0.10 (0.01 to 0.20)	0.04
Attitude towards partner notification	11.59 (1.74)	11.63 (1.74)	−0.04 (−0.14 to 0.05)	0.37
Self-efficacy in telling partner about an infection	11.55 (3.80)	11.53 (3.90)	0.04 (−0.17 to 0.24)	0.72
Correct condom use self-efficacy	14.57 (2.90)	14.27 (2.97)	0.32 (0.16 to 0.47)	<0.001
Self-efficacy in negotiating condom use	11.35 (2.50)	11.32 (2.60)	0.03 (−0.10 to 0.17)	0.64
**Intermediate outcomes (structural equation model)†**				
Knowledge related to STIs	-	-	0.081	0.02
Attitude towards partner notification	-	-	0.031	0.39
Self-efficacy in telling partner about an infection	-	-	0.020	0.55
Correct condom use self-efficacy	-	-	0.118	<0.001
**Sharing of intervention content**	n=2414	n=2453		
Participant knew someone taking part in the study:	137 (5.7)	141 (5.8)		
They read participant’s messages	37 (1.5)	32 (1.3)		
Participant read their messages	38 (1.6)	34 (1.4)	-	-
**Reading intervention content**	n=2416			
Did anyone read the messages sent to you?:	342 (14.2)	NA		
Yes	1971 (81.6)	NA		
No or unsure	103 (4.3)	NA	-	-
How did you feel about them reading the messages?:	n=342			
Happy	224 (65.5)	NA		
Unhappy	35 (10.2)	NA		
Unsure	83 (24.3)	NA		
How many of the messages did you read?:	n=2412			
All	1506 (62.4)			
Most	661 (27.4)			
Few	229 (9.5)			

STI=sexually transmitted infection; NA=not applicable.

* Regression of summed items, adjusted for same baseline characteristics as primary analysis: age, ethnicity, type of infection at baseline, sexual orientation group. Ranges of possible scores: Knowledge 3-15; attitude towards partner notification 3-15; self-efficacy in telling a partner about an infection 4-20; correct condom use self-efficacy 4-20; self-efficacy in negotiating condom use 3-15.

† Results from structural equation model (using latent variable process outcomes). Coefficients are standardised so that the interpretation is that compared with the control group, the intervention group has 0.081 standard deviations greater knowledge related to STIs. Adjusted for same baseline characteristics as primary analysis: age, ethnicity, type of infection at baseline, sexual orientation group.

Similar findings to those of the main analysis were obtained from additional sensitivity analyses that were not prespecified (see appendix 2). Analyses were undertaken under different assumptions from those of the primary analysis missing-at-random assumption and included a post hoc analysis adding baseline number of partners (<2 or ≥2 partners) to the imputation model as an additional covariate for the primary outcome and the outcome number of partners. An additional analysis found the number need to harm was 64 (95% confidence interval number needed to benefit 334 to ∞ to number need to harm 24).

## Discussion

Our text messaging intervention (safetxt) targeting partner notification, condom use, and STI testing did not reduce the risk of chlamydia or gonorrhoea reinfection at one year. More infections occurred in the safetxt intervention group compared with control group that only received text messages to query any changes to postal or email address. Some increase was found in self-reported precautionary behaviours such as condom use, but the number of STIs was not reduced. Although our intervention did not target sexual partnerships, the proportion of people with a new partner and with two or more partners at one year was higher in the intervention group.

### Strengths and limitations of this study

We ensured allocation concealment by using a web based randomisation system. Baseline prognostic factors were well balanced between the two groups. Data collectors, laboratory analysts, and statistical analysts were masked to treatment allocation. Chlamydia and gonorrhoea were diagnosed using nucleic acid amplification polymerase chain reaction tests with high sensitivity and specificity. The primary analyses were on an intention-to-treat basis. Our recruitment across sociodemographic groups and sexualities, with no evidence of heterogeneity of effects in subgroups, suggests the results are generalisable.

Our trial has some limitations. A high proportion of eligible people declined to participate in the study. Many were only approached by text message or email and did not respond. Compared with the general UK population, those living in areas with a high index of multiple deprivation (fourth and fifth fifths) and ethnic minorities were well represented in our trial. Although 2162 men took part, men were under-represented and women over-represented in the trial. The primary outcome was only available for 75% of participants in each group. Although we used evidence based methods to achieve higher follow-up for laboratory assessed chlamydia and gonorrhoea than previous similar trials,^19^ some potential for bias remains. In our primary analysis we used multiple imputation methods to reduce bias and increase precision of the effect estimates.^5^ ^15^ The similarity of findings in our primary analysis and all sensitivity analyses is reassuring. Many of our secondary outcomes were self-reported and could be influenced by social desirability bias.

### Comparison with other studies

Our trial explored the effects of safer sex text message support on objectively measured STI outcomes.^20^ ^21^ In previous trials the effects of interventions delivered by text message on condom use in the long term (12 months) were uncertain (pooled effect estimate relative risk 1.10, 95% confidence interval 0.77 to 1.56, I^2^=7%; three trials, n=667) and at risk of bias from incomplete follow-up, selection bias in a cluster randomised controlled trial, and lack of blinding of those collecting follow-up data.^8^ ^20^ ^21^ ^22^ ^23^ The effects of our text message intervention on condom use at one year are modest (odds ratio 1.14, 95% confidence interval 1.01 to 1.28, P=0.038) but larger than those reported for single sessions of face-to-face counselling or other forms of remote support such as telephone counselling, videos, or websites at 12 months.^24^ ^25^

In a recent systematic review, the pooled odds ratio for text messages on STI testing within 12 months was 1.83 (95% confidence interval 1.41 to 2.36; seven trials, n=2151) (moderate certainty evidence).^20^ Our trial found no benefit on STI testing before sex with new partners. Before the main and pilot trial of safetxt, a Ugandan trial had reported an odds ratio of 1.54 (95% confidence interval 0.85 to 2.79) for the effect of text messages on partner attendance for syphilis testing and treatment at next antenatal care visit.^20^ ^21^ ^26^

The effects of text messages in promoting sexual health might be context and content specific. The effect on STIs of digital messages targeting condom use and STI testing only among those reporting risky behaviour but with no STI diagnosed remains uncertain.

Our intervention was not associated with a reduction in risk of chlamydia or gonorrhoea at one year and could have increased risk. We explored methodological reasons for the unexpected results. Firstly, we conducted analyses using a range of different assumptions about STI rates in those lost to follow-up in both groups. An active care seeking effect resulting in more people with STI in the intervention group being identified is unlikely as follow-up for the primary outcome was similar in both groups (slightly higher in the control group), and the results of sensitivity analysis testing assumptions that the outcome data are not missing at random provided results that were similar to the findings of our main trial analysis (see appendix 2). Secondly, we explored if these findings could have occurred as a result of small chance imbalances in sexual behaviour (number of partners in preceding 12 months) between the intervention group and control group at baseline. The consistency of the results of all these analyses combined with the slightly increased effect size in the per protocol analysis adds to the weight of evidence suggesting our intervention increased the risk of STIs. When we pooled the results of the trial with data from pilot trial participants receiving the same intervention targeting partner notification, condom use, and STI testing, risk of reinfection remained similar (pooled odds ratio 1.12, 95% confidence interval 0.99 to 1.26, P=0.08, I^2^=0%, see supplementary figure S1).

Sexual behaviour is complex, and a review of our qualitative research findings and open feedback comments provided by participants raises a potential mechanism for the slightly higher number of infections in the intervention group.^9^ This concerned a reduction in felt stigma (internal stigma or self-stigmatisation) in having an STI, leading to the intervention group having more partners than the control group and hence more STIs. To encourage partner notification, the intervention adopted a non-stigmatising approach to providing information and support. Recipients reported a reduction in felt stigma about having an STI as a benefit of the intervention.^9^ Lower levels of stigma are associated with higher precautionary and treatment behaviours (eg, condom use, STI testing, and STI treatment), improved emotional and mental wellbeing, lower pregnancy rates in adolescents, and also higher numbers of sexual partners.^27^ ^28^ ^29^ Lower stigma measured at a societal level is associated with higher country level STIs.^30^ If our intervention reduced felt stigma about having an STI, this could have resulted in recipients having more partners than the control group leading to a higher risk of STIs. Some group based behavioural interventions have been associated with increased numbers of STIs, thought to be due to the aggregation of at risk individuals.^31^ Although in this trial we did not introduce participants to each other, they reported a reduced sense of isolation in having an STI—realising they were “not the only one.”

Data from our trial show that between baseline and 12 month follow-up self-reported condom use at last sex increased in both groups (23.9% intervention and 25.8% control; 33.8% intervention and 31.2% control, respectively), and the number of participants who reported having two or more partners was noticeably reduced (83.9% intervention and 82.7% control; 56.9% intervention and 54.8% control, respectively). Large behavioural changes occurred among the participants in our trial who had a diagnosis of an STI, received current care pathways in community sexual and reproductive health clinics or genitourinary clinics, and may have accessed information currently available in the public domain. Compared with this finding, the effect of text messages was small. Safer sex behaviours are influenced by a wide range of individual, interpersonal, societal, and structural factors, which could also explain why the intervention effects were small.

### Policy implications

Our qualitative research suggests that from the perspective of young people, the safetxt intervention positively impacts on broader aspects of sexual and reproductive wellbeing, such as confidence, agency, and communication about sexual health with siblings, friends, and partners.^9^ In this trial our intervention showed benefits on some measures of sexual health, such as self-efficacy in condom use and condom use in itself, whereas the intervention’s public health effects on STIs were in the direction of harm. This illustrates the importance of rigorously evaluating the impact of novel health communication interventions on objective public health outcomes.

It is not likely that the approaches to promoting condom use resulted in increased numbers of STIs, as these methods were adapted from, and similar to, the content of face-to-face interventions that have been shown to increase condom use and reduce the number of STIs.^32^ ^33^ ^34^

Our text message intervention was grounded in psychological theory, incorporating the best evidence on health behaviour change, but it did not have the effects we anticipated. In light of our results, WHO should revise its endorsement of digital behaviour change communication for strengthening health systems, to specify which topics and content WHO endorses.

### Conclusions

Safetxt did not reduce STIs. More reinfections occurred in the intervention group. Our results highlight the need for rigorous evaluation of health communication interventions. Future work could evaluate the effect of interventions promoting condom use and STI testing in those at risk but with a diagnosis of an STI. Further research should focus on how to reduce the stigma associated with STIs to benefit wellbeing, treatment, and precautionary behaviours for those with a diagnosis of an STI, without increasing the risk of infection.

**What is already known on this topic**

Behaviour change interventions delivered by automated text message (such as for smoking cessation) can be highly cost effective
A review on the effects of sexual health interventions delivered by text message found little high quality evidence
The effects on key behaviours such as condom use, partner notification, and outcomes of sexually transmitted infections (STIs) were uncertain

**What this study adds**

The safetxt intervention using a mobile phone and targeting safer sex behaviours was not associated with a reduction in incidence of chlamydia or gonorrhoea at one year; more infections occurred in the intervention group
Saftext was associated with an increase in some self-reported measures of sexual health, such as self-efficacy in condom use and condom use in itself
WHO should revise its endorsement of digital behaviour change communication for strengthening health systems, to specify which topics and content WHO endorses

## Data Availability

After publication of the primary and secondary analyses detailed in the statistical analysis plan, individual deidentified patient data, including a data dictionary, will be made available via our data sharing portal FreeBIRD website indefinitely. The trial protocol, statistical analysis plan, and trial publications will be freely available online.

## Web extra

Extra material supplied by authors

Supplementary material: Appendices 1 and 2, including supplementary tables S1-S3, figure S1, and additional information

## Notes

Contributors: CF, MJP, OLM, IR, LJ, SB, and KP wrote the report with comments from KT, LM, KD, MK, JRC, TPM, ZJ, FD. RF, FCIH, AG, KW, PB, JB, TC, PE, and GH. SM, CF, and PB conceptualised the intervention. CF, OLM, RSF, FH, PB, KW, JVB, GH, SM, and KD designed the intervention. CF, OLM, TC, PE, and IR designed the trial. TPM, JRC, and PE were trial statisticians. TPM, JRC, and MJP conducted the trial analysis. MJP conducted the analysis of process measures. SB conducted the pooled analyses of data. KP, OLM, LJ, MJP, MK, and ZJ were responsible for project administration. OLM, LJ, MK, MJP, KP, FD, ZJ, and CF developed the trial recruitment and follow-up procedures. KP was responsible for the day-to-day running of the trial. LJ, MK, and MJP assisted recruitment. LJ, MK, ZJ, and FD undertook trial follow-up. MJP, TPM, and JRC prepared figures and tables. CF, OLM, RSF, FCIH, PB, KW, GH, SM, KD, PE, IR, and GP contributed to writing the grant application. CF is the guarantor and accepts full responsibility for the work and the conduct of the study, had access to the data, and controlled the decision to publish. The corresponding author (CF) attests that all listed authors meet authorship criteria and that no others meeting the criteria have been omitted.

The lead author (CF) affirms that the manuscript is an honest, accurate, and transparent account of the study being reported; that no important aspects of the study have been omitted; and that any discrepancies from the study as planned (and, if relevant, registered) have been explained.

Dissemination to participants and related patient and public communities: The results will be emailed to the participants who requested them and will be disseminated to all participating clinics. Patient representatives are developing a dissemination plan that they will be actively involved in, including dissemination via social media.

Provenance and peer review: Not commissioned; externally peer reviewed.
