# Supplementary material for: Effectiveness of a behavioural intervention delivered by text messages (safetxt) on sexually transmitted reinfections in people aged 16-24 years: randomised controlled trial
Source: BMJ. 2022 Sep 28;378:e070351. doi: 10.1136/bmj-2022-070351 (PMC9516322; doi:10.1136/bmj-2022-070351)

Appendix 1: Example text messages for those diagnosed with chlamydia

Treatment and 7 days abstinence after treatment

Day 1 You made the right decision to get a test. Getting treated quickly means you are less likely to have any problems.

Chlamydia is a common bacterial infection that's easy to treat with antibiotics. To treat the infection, take the tablets and then don't have sex (oral, vaginal and anal)
for 7 days while the infection clears.

Day 2 It's common to get re-infected with chlamydia. To avoid getting it again, the next steps are: Day 1) get treated Day 2) tell the person you're having sex with to get treated 3) don't have sex for 7 days (oral, vaginal or anal) after you and your partner(s) have been treated.

Telling partner (after initial diagnosis)

Day 1 Most people who have an infection don't know. Your partner(s) could be infected so it's important to tell them that they need treatment too.

Here are a few examples of how others told their partner: "I said 'I don't really want to tell you this but I have to- I found out I have chlamydia.'

It's awkward to tell people but it's not right not to, is it? They may not know. You can't just let them walk round with an infection." Text 1 to hear more.

Preventing re-infection, info on specific STI (depending on type of STI participants had at baseline)

Day 6 Most people who have an infection don't know. You can't tell if someone has an infection just by looking at them or by how well you know them.

Day 10 Some people say they didn't use a condom because their partner didn't want to use one. Here are some examples of how other people convinced their partner to use one: "I said using a condom was about respecting each other." Text 14 to hear more.

If texted 14 -"I explained that it's not them that I don't trust, it's the people that they've been with before."

Day 11 A lot of the time, sex isn't planned. So it's best to always have a condom on you. Find a time to put a few in your wallet. You could also keep a supply in places where you have sex (bedroom, partner's house, car).

Day 17 One reason a condom may split is because there is air trapped inside. To

	prevent this, hold the tip of the condom between your forefinger and thumb and roll it down, making sure there are no air bubbles.
**Day 40**	When you just start seeing someone, it can be awkward to bring up condoms. Most people are happy to talk about condoms though.<br>More than likely they're thinking the same thing and will be relieved that you brought it up first. It can help to think about what you'll say beforehand.

Example control group message:

**Day 60-**	Hi, it's Ona here. Thank you for taking part in the texting study. Remember to let us know if your contact details have changed by replying to this text or emailing safetxt@lshtm.ac.uk

Appendix 2 – Additional information and supplementary tables and figure

Analysis of the intermediate outcomes

The intermediate outcome measure comprised multiple ordinal scales. Using data from the first 1025 randomised participants, we assessed the construct validity of the intermediate outcomes and refined them using confirmatory factor analysis (CFA). The originally specified CFA model was based on the a priori factor structure of the model (which items loaded on which factors), as shown in Table S1. For this original model, the goodness of fit indices indicated borderline fit (. root mean square error of approximation RMSEA: 0.083; comparative fit CFI 0.936; Tucker-Lewis index TLI: 0.923). After examining the modification indices to identify sources of poor fit, the model was revised. The variable "most people who have an STI will tell their partner" was dropped from the model due to having a low factor loading of 0.287 on attitudes to partner notification. The variable relating to how easy or difficult it would be to "Put a condom on" was dropped due to cross loadings (indicating a lack of discriminant validity) between the 'Correct condom use self-efficacy' factor and the 'Self–efficacy in negotiating condom use' factor. Finally, we allowed the error terms of the variables "How easy or difficult would it be to tell the last person you had sex with that you had an STI" and "How easy or difficult would it be to tell the last person you had sex with to get treatment" to correlate; and did the same for the equivalent variables that referred to a 'new partner'. We considered this appropriate given that the correlations of error terms between these pairs of variables is likely to be a case of an 'item priming effect'[65]. It seems reasonable that the answer to the first question in each of these pairs will directly affect

how the respondent answers the ‘treatment’ item, as informing a partner of one’s infection is a prerequisite to informing them that they will need treatment. Once these changes had been applied, the revised model showed good fit to the data (RMSEA: 0.052, CFI: 0.980, TLI: 0.975). Furthermore, multi-group analyses across genders, sexual orientation, and mode of questionnaire (phone versus written) indicated measurement equivalence across these groups.

The impact of the intervention of these refined intermediate outcome measures was examined. To aid interpretability, we present the results of two analyses. One is based on summing the responses to each item contributing to that intermediate measure, and using a linear regression to test for a difference in mean scores between the arms. The second analysis extends the CFA measurement model described above into a structural equation model, using the allocation as the main predictor variable, thereby estimating the impact of the intervention on the intermediate outcomes in the absence of measurement error. These regressions were adjusted for the same covariates as the primary analyses.

Intermediate outcome results

The effects of the intervention on the intermediate outcomes (measured by summing items) are reported in Table 3 in the paper. Table S2 presents the results of the structural equation modelling, estimating the impact of the intervention on the intermediate outcomes in the absence of measurement error. The results are consistent with those presented in Table 3, with the intervention resulting in a small increase in knowledge related to STIs and in correct condom use self-efficacy.

Table S1. Intermediate outcomes and corresponding questionnaire items

Theoretical construct	Question Item	Answer Options
**Knowledge related to STIs**	**To what extent do you agree or disagree with the following:**	
	If someone had a sexually transmitted infection (STI), they would know.	1. Strongly disagree<br>2. Disagree<br>3. Unsure<br>4. Agree<br>5. Strongly agree
	STIs are rare.	As above
	I can tell if someone has an STI.	As above
**Attitudes towards partner notification**	**To what extent do you agree or disagree with the following:**	
	Most people who have an STI will tell their partner.[a]	As above
	It's my responsibility to tell a partner if I get diagnosed with an STI.	As above
	If I tell my partner I have an STI, my partner would be glad I let them know.	As above
	If I tell my partner I have an STI, my partner would think badly of me.	As above
**Self–efficacy in telling a partner about an infection**	**How easy or difficult would it be to:**	
	Tell the last person you had sex with that you had an STI	1. Very easy<br>2. Easy<br>3. Unsure<br>4. Difficult<br>5. Very difficult
	Tell the last person you had sex with to get treatment	As above
	Tell a new partner you had an STI	As above
	Tell a new partner to get treated	As above
**Correct condom use self-efficacy**	**How easy or difficult would it be to:**	
	Put a condom on [a]	As above
	Keep a condom from drying out during sex	As above
	Keep a condom from breaking or coming off during sex	As above
	Keep a condom on while withdrawing the penis	As above
	Keep a condom on from start to finish	As above
**Self–efficacy in negotiating condom use**	**How easy or difficult would it be to:**	
	Imagine that you and your partner have sex but don't use condoms. You want to start using condoms. How easy or difficult would it be for you to tell your partner that you want to use condoms?	As above
	Imagine that you are having sex with someone new. You want to use condoms. How easy or difficult would it be for you to tell them that you want to use condoms?	As above
	Imagine that you are having sex with someone new. You want to	As above

	use condoms. How easy or difficult would it be for you to tell them that you won't have sex unless you use condoms?	
[a] Variable dropped from the model, as explained in text ('Analysis of the intermediate outcomes' section).		

Table S2. Intermediate outcomes comparing the intervention group to the control group (structural equation model)

	Coefficient (beta)*	p-value
**Intermediate outcomes**		
Knowledge related to STIs	0.081	0.021
Attitudes towards partner notification	0.031	0.388
Self–efficacy in telling a partner about an infection	0.020	0.549
Correct condom use self-efficacy	0.118	<0.001
Self–efficacy in negotiating condom use	0.000	0.996
*Complete case analysis results from structural equation model (using latent variable intermediate outcomes). Coefficients are standardized, so that the interpretation is follows: compared to the control group, the intervention group has 0.081 standard deviations greater knowledge related to STIs. Adjusted for same baseline characteristics as primary analysis: age, ethnicity, type of infection at baseline, sexuality group.		

Trial -Additional Sensitivity analyses

We performed additional non-pre-specified sensitivity analyses under different assumptions from the primary analysis MAR assumption. Sensitivity analysis 1: We completed the MI model including the clinic testing variable an additional covariate. On this imputed dataset, we conducted one sensitivity analysis with the new imputations from this model where all negative clinic tests who have missing outcome data were considered positive. The result from this analysis was OR 1.13 (95% CI 0.997 to 1.28, p=0.05). Sensitivity analysis 2: Using the same imputed dataset (with the clinic testing variable as an additional covariate), we conducted a second sensitivity analysis where all negative clinic tests who had missing outcome data were considered negative. The result from this analysis was OR 1.12 (95% CI 0.97 to 1.29, p=0.13). Sensitivity analysis 3. Sensitivity analysis 3. We followed the primary analysis that assumed missing at random but in imputing missing values, controlled the odds of STI diagnosis to be ¼, ½, 1, 2, and then 4 times as large as that predicted by the imputation model; these sensitivity parameters were varied factorially for the two randomised groups (giving 24 sensitivity scenarios besides the primary analysis). The results were identical to the primary outcome result: OR 1.13, 95% CI 0.98 to 1.31, p= 0.085. This was due to 1)

perfect prediction in the imputation model and 2) using the same random number seed to start each sensitivity analysis.

Including baseline number of partners in the imputation model.

We conducted a post hoc analysis replicating the analysis but adding baseline number of partners (< or ≥2 partners) to the imputation model as an additional covariate for both the primary outcome and for the outcome number of partners. The OR for cumulative incidence of chlamydia/gonorrhoea for this analysis was 1.13 (95% CI 0.98-1.31, p=0.087). The OR for number of partners was 1.10 (0.98 - 1.23; p=0.11).

Per protocol analysis

We conducted a per protocol analysis where participants who had 12 month primary outcome data were classified as having received the treatment they were allocated to according to the following criteria: 1) they did not stop the messages; 2) they were not among the few participants that did not receive any messages and 3) they reported that they read all or most of the messages. Baseline characteristics among these participants were similar between the groups (Table S3). The OR for cumulative incidence of GC/CT for this analysis was 1.17 (95% CI 0.99-1.38, p=0.06).

Pooled analysis with the safetxt pilot trial data.

We conducted a pooled analysis with all the main trial and pilot trial data from participants diagnosed with an STI at baseline (where the intervention group had been allocated to receive content targeting partner notification, condom use and STI testing. The pooled odds ratio was 1.12 (95% CI 0.99- 1.26), P=0.08, I^2=0% (figure S1).

Table S3: Description of participants in the per-protocol population

	Intervention N=2019, n (%)	Control N=2229, n (%)	Total N=4248, n (%)
**Age group**			
16-19	778 (38.5%)	799 (35.8%)	1577 (37.1%)
20-24	1241 (61.5%)	1430 (64.2%)	2671 (62.9%)
**Gender**			
Female	1398 (69.2%)	1506 (67.6%)	2904 (68.4%)
Male	614 (30.4%)	717 (32.2%)	1331 (31.3%)
Non-binary gender	7 (0.3%)	6 (0.3%)	13 (0.3%)
**Ethnicity grouped**			
White British/ Other White background	1618 (80.1%)	1780 (79.9%)	3398 (80.0%)
Black/Black British - Caribbean/African/other	219 (10.8%)	223 (10.0%)	442 (10.4%)
Asian/Asian British - Bangladeshi/Chinese/Indian/ Pakistani/other	46 (2.3%)	59 (2.6%)	105 (2.5%)
Mixed background	104 (5.2%)	140 (6.3%)	244 (5.7%)
Other background	32 (1.6%)	27 (1.2%)	59 (1.4%)
**Educational level**			
16 or under	283/2000 (14.2%)	298/2199 (13.6%)	581/4199 (13.8%)
17 or over	861/2000 (43.1%)	997/2199 (45.3%)	1858/4199 (44.2%)
I am still in full time education	856/2000 (42.8%)	904/2199 (41.1%)	1760/4199 (41.9%)
**Gender and orientation**			
WSM	1296 (64.2%)	1375 (61.7%)	2671 (62.9%)
MSW	429 (21.2%)	485 (21.8%)	914 (21.5%)
WSW	16 (0.8%)	12 (0.5%)	28 (0.7%)
MSM	152 (7.5%)	186 (8.3%)	338 (8.0%)
WSWM	85 (4.2%)	118 (5.3%)	203 (4.8%)
MSWM	33 (1.6%)	46 (2.1%)	79 (1.9%)
NBSM	5 (0.2%)	1 (0%)	6 (0.1%)
NBSW	0	2 (0.1%)	2 (0%)
NBSWM	2 (0.1%)	3 (0.1%)	5 (0.1%)
not stated	1 (0%)	1 (0%)	2 (0%)
**Baseline diagnosis**			
Chlamydia	1606 (79.5%)	1758 (78.9%)	3364 (79.2%)
Gonorrhoea	182 (9.0%)	217 (9.7%)	399 (9.4%)
Gonorrhoea and Chlamydia	97 (4.8%)	110 (4.9%)	207 (4.9%)
Gonorrhoea or NSU	17 (0.8%)	18 (0.8%)	35 (0.8%)
NSU (non-specific urethritis)	71 (3.5%)	77 (3.5%)	148 (3.5%)
Unknown	46 (2.3%)	49 (2.2%)	95 (2.2%)
**Baseline condom used last sex**			
Yes	500 (24.8%)	569 (25.5%)	1069 (25.2%)
No	1486 (73.6%)	1624 (72.9%)	3110 (73.2%)
Unsure	33 (1.6%)	36 (1.6%)	69 (1.6%)
**Baseline condom used new partner**			
Yes	653 (32.3%)	730 (32.8%)	1383 (32.6%)
No	1325 (65.6%)	1448 (65.0%)	2773 (65.3%)
Unsure	41 (2.0%)	51 (2.3%)	92 (2.2%)
**Baseline tested before sex new partner**			
Yes	787 (39.0%)	900 (40.4%)	1687 (39.7%)
No	1174 (58.1%)	1263 (56.7%)	2437 (57.4%)

	**Intervention** N=2019, n (%)	**Control** N=2229, n (%)	**Total** N=4248, n (%)
Unsure	58 (2.9%)	66 (3.0%)	124 (2.9%)
**Baseline partner tested before sex new partner**			
Yes	282/2018 (14.0%)	321 (14.4%)	603/4247 (14.2%)
No	773/2018 (38.3%)	836 (37.5%)	1609/4247 (37.9%)
Unsure	963/2018 (47.7%)	1072 (48.1%)	2035/4247 (47.9%)
**Baseline number of partners** 0	4/2018 (0.2%)	2/2227 (0.1%)	6/4245 (0.1%)
1	310/2018 (15.4%)	372/2227 (16.7%)	682/4245 (16.1%)
2+	1704/2018 (84.4%)	1853/2227 (83.2%)	3557/4245 (83.8%)
Data are n (%), mean (SD), or n/N (%). WSM (women who have sex with men only), MSW (men who have sex with women only), WSW (women who have sex with women only), MSM (men who have sex with men only), WSMW (women who have sex with men and women), MSWM (men who have sex with women and men), NBSM (non-binary people who have sex with men only), NBSW (non-binary people who have sex with women only), NBSWM (non-binary people that who have sex with women and men);			

Fig S1. Cumulative incidence of gonorrhoea or chlamydia infection (objectively assessed at 12 months) among participants diagnosed with an STI at baseline.

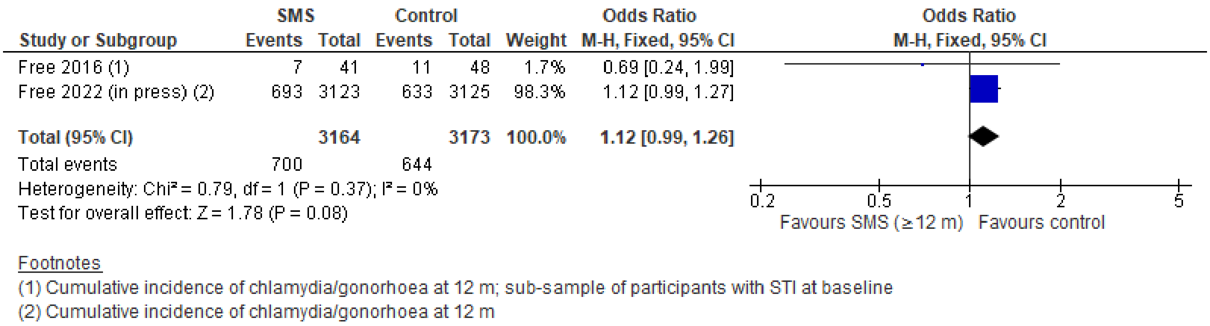

Supplement: Supplementary file 1 — Supplementary material: Appendices 1 and 2, including supplementary tables S1-S3, figure S1, and additional information [file frec070351.ww.pdf]
